# Association of tea and coffee consumption with the risk of all-cause and cause-specific mortality among individuals with metabolic syndrome: a prospective cohort study

**DOI:** 10.1186/s13098-023-01222-7

**Published:** 2023-11-23

**Authors:** E Wu, Ying-Ying Bao, Guo-Fang Wei, Wei Wang, Hong-Quan Xu, Jia-Yin Chen, Ya-Nan Xu, Dan Han, Lin Tao, Jun-Tao Ni

**Affiliations:** 1 Rehabilitation and Nursing School, Hangzhou Vocational & Technical College, Hangzhou, Zhejiang 310018 China; 2 School of Pharmacy, Hangzhou Normal University, Hangzhou, Zhejiang 311121 China; 3 Key Laboratory of Elemene Class Anti-Cancer Chinese Medicines, Engineering Laboratory of Development and Application of Traditional Chinese Medicines, Collaborative Innovation Center of Traditional Chinese Medicines of Zhejiang Province, Hangzhou Normal University, Hangzhou, Zhejiang 311121 China; 4 Women’s Hospital School of Medicine Zhejiang University, Hangzhou, Zhejiang 310006 China

**Keywords:** Coffee, Tea, Metabolic syndrome, All-cause mortality, Cause-specific mortality

## Abstract

**Background:**

The relationship between tea and coffee consumption and mortality among patients with metabolic syndrome (MetS) remains barely explored. Herein, this study aimed to examine the association between tea and coffee consumption and the likelihood of all-cause and cause-specific mortality in patients with MetS.

**Methods:**

A total of 118,872 participants with MetS at baseline from the UK Biobank cohort were included. Information on tea and coffee consumption was obtained during recruitment using a touchscreen questionnaire. Hazard ratios (HRs) and 95% confidence intervals (CIs) for mortality were determined using Cox proportional hazards models.

**Results:**

During a median follow-up of 13.87 years, 13,666 deaths were recorded, with 5913, 3362, and 994 deaths from cancer, cardiovascular diseases (CVD), and respiratory disease (RD), respectively. This research showed a significant inverse association between tea intake and the risk of all-cause and cancer mortality, the respective HRs (95% CI) for consuming tea 2 vs. 0 cup/day were 0.89 (0.84–0.95), and 0.91 (0.83–0.99), and tea intake ≥ 4 cups/day could reduce CVD mortality by 11% (HR 0.89; 95% CI 0.81–0.98). The U-shaped nonlinear association between coffee intake and all-cause/CVD mortality was examined (all *p-*nonlinear < 0.001). The HRs (95% CI) for coffee consumption 1 vs. 0 cup/day were 0.93 (0.89–0.98) and 0.89 (0.80–0.99), and for ≥ 4 vs. 0 cup/day were 1.05 (1.01–1.11) and 1.13 (1.03–1.25), respectively. Notably, the combined intake of tea and coffee presented a protective effect against all-cause mortality (HR < 1).

**Conclusions:**

The importance of daily tea and moderate coffee consumption in individuals with MetS to optimise health benefits are highlighted.

**Supplementary Information:**

The online version contains supplementary material available at 10.1186/s13098-023-01222-7.

## Background

Metabolic syndrome (MetS) is a complex condition characterised by a cluster of interrelated metabolic abnormalities and encompasses a range of risk factors, such as insulin resistance, hypertension, abdominal obesity, and dyslipidaemia [1]. The global incidence of MetS has risen rapidly, affecting an estimated one-quarter of the global population in 2018 [2]. The detrimental effects of MetS on human health must not be neglected. Patients with MetS have a 50% higher risk of death and a significantly increased risk of developing complications such as cardiovascular diseases (CVD) and cancer [3–5]. Moreover, MetS poses a substantial economic burden on society due to increased healthcare costs, decreased productivity, and lost workdays [6]. Given the increasing burden of MetS due to factors such as an aging global population, it is imperative to identify risk factors, especially modifiable dietary factors, to aid the development of effective prevention and management strategies.

Recently, there has been a growing focus on the impact of diet on the development and management of MetS [7]. Among the numerous dietary components, coffee and tea have gained particular attention because of their potential influence on metabolism [8, 9]. Tea and coffee, two beverages widely consumed worldwide, are also recognised for their bioactive compounds, such as caffeine and polyphenols, which are known to have various physiological effects on human metabolism, including improved insulin sensitivity, reduced inflammation, and protection against CVD [10, 11]. Studies have suggested that regular consumption of tea and coffee may be linked to a lower risk of developing MetS and its components [8, 12]. While previous studies have explored the association between tea and coffee consumption and mortality [13, 14], limited evidence specifically focuses on patients with MetS.

The main objective of this study is to address this lack of knowledge by conducting a prospective cohort study using data from the UK Biobank. This study aimed to examine the relationship between tea and coffee consumption and the risk of all-cause and cause-specific mortality in patients with MetS. The results of this study have significant implications for public health and contribute to dietary recommendations for preventing and managing mortality in patients with MetS.

## Materials and methods

### Study participants

The UK Biobank is a ground-breaking cohort research initiative aimed at improving the understanding of human health and diseases. It collected abundant data and biological samples from approximately half a million individuals aged 37–73 years in the UK (2006–2010) and followed them continuously. The volunteers provided detailed information on their medical history, lifestyle habits, and environmental exposures at one of the 22 assessment centres in the UK and generously donated blood, urine, and saliva samples for analysis [15]. For the current study, participants who had missing information on tea and coffee consumption, were < 40 or > 70 years old, and did not suffer from MetS at recruitment were excluded, as were those who were lost to follow-up or died within the first 3 follow-up years. Finally, 118,872 participants were included in the analysis (Supplementary Fig. S1).

### Ascertaining MetS

The clinically diagnosing of MetS typically involves the presence of three or more of the following criteria [1]: ① abdominal obesity: determined by waist circumference according to ethnic and sex-specific criteria; ② high blood pressure (BP): BP ≥ 130/85 mmHg, or antihypertensive therapy; ③ Elevated fasting glucose (FG): FG ≥ 100 mg/dL, or medication for elevated glucose; Of note, the UK Biobank participants were examined for blood biochemistry at random and in a non-fasting state, and we used glucose ≥ 11.1 mmol/L (200 mg/dL) or HbA1c ≥ 48 mmol/mol (6.5%) range to indicate hyperglycaemia even in the non-fasting state [16, 17]; ④ Lower high-density lipoprotein cholesterol (HDLC): HDLC < 1.0/1.3 mmol/L in males/females or drug therapy for lower HDLC; ⑤ High triglycerides (TG): TG ≥ 1.7 mmol/L or medication for high TG.

### Exposure assessment

Information on tea and coffee consumption were obtained using a touchscreen questionnaire at the time of recruitment. Tea/coffee consumption: “How many cups of tea/coffee do you drink each day?“ (Field ID:1488 and 1498); The participants were asked to confirm if the answer was > 20/10 for tea/coffee consumption. We recorded consumption below 1 cup/day as zero, and other answers < 0 or > 99 were considered missing values [18].

### Ascertainment of outcomes

The primary outcome was mortality during the follow-up period. Details on mortality were obtained from the death register, which was linked to the NHS Digital Centre (England/Wales) and the NHS Central Register (Scotland). The cause-specific mortality included cancer, CVD, and RD (Supplementary Table S1) [19]. Person-time was computed from baseline to the date of death or 30 November 2022 (whichever came first).

### Assessment of covariates

The selection of covariates was based on prior knowledge and previous research [14], including sociodemographic characteristics (age, sex, ethnicity, education, and socioeconomic status); lifestyles (body mass index [BMI], diet, smoking, drinking, and exercise frequency); medication and disease history (cholesterol-lowering medication, BP medication, insulin, and depression). All covariates were collected at baseline, and a more comprehensive description of the covariates is provided in Supplementary Methods S1.

### Statistical analysis

The participants’ baseline characteristics were summarised according to coffee and tea consumption categories. Categorical variables are presented as frequency (percentages), while continuous variables are presented as mean (standard deviation [SD]) (normal distribution) or median (interquartile range [IQR]) (non-normal distribution). Associations between tea and coffee consumption and baseline characteristics were determined using generalised linear models. The reverse Kaplan-Meier method was used to determine the median person-years of follow-up. For continuous variables, missing values were replaced with median values, whereas for categorical variables, missing values were replaced with mode values (Supplementary Methods S2) [19].

Cox proportional hazards regression models were used to analyse the association between tea and coffee consumption and mortality in patients with MetS. We adjusted for sociodemographic characteristics in Model one, including age, sex, ethnicity, education, and socioeconomic status; and we further adjusted for lifestyle factors (BMI, diet, smoking, drinking, and exercise frequency) in model two; and we additionally controlled for medication and disease history (cholesterol-lowering medication, BP medication, insulin, and depression) in model three. Notably, tea or coffee consumption was additionally adjusted for coffee or tea analysis. Schoenfeld residuals were used to test the proportional hazards assumption and no substantial evidence of non-proportionality was found (*p* > 0.05). The dose-response relationship between separate or combined tea and coffee intake and death risk was analysed using multivariate-adjusted restricted cubic splines (RCS). Separate tea and coffee consumption was divided into five groups: 0, 1, 2, 3, and ≥ 4 cups/day. Additionally, stratified analyses were conducted to investigate whether the relationship between tea and coffee consumption and the likelihood of death differed according to baseline factors, such as age, sex, and lifestyle.

Sensitivity analyses were performed to verify the robustness and validity of the results. First, considering the difficulty in assessing the role of depression in these associations, we repeated the primary analysis without adjusting for depression at recruitment. Second, we excluded participants with significant pre-existing conditions (CVD and cancer) at recruitment to minimise reverse causality (*n* = 98,102). Statistical analyses were performed using R (version 4.1.3) and Stata 15.0. Statistical significance was set at *p* < 0.05 (two-sided) was used to determine statistical significance.

## Results

### Baseline characteristics

A total of 118,872 participants with MetS (median [IQR] age: 59 [12]; 50.5% female) were included in the analysis. Among them, 92.8% were of white ethnicity, and 43.1% and 21.2% reported drinking tea or coffee ≥ 4 cups/day, respectively. Compared to non-tea drinkers, those who drank tea ≥ 4 cups/day tended to be older, male, had a better socioeconomic status, had a lower BMI, never smoked, exercised more frequently, and had a healthy diet. Similarly, compared to non-coffee drinkers, those who consumed coffee ≥ 4 cups/day tended to be older white males, had higher education, and had a better socioeconomic status (Table 1). Supplementary Table S2 presents the baseline characteristics of the combined distributions of tea and coffee consumption.

**Table 1:** Baseline characteristics by coffee and tea intake

	Tea intake, cups/day, No. (%)						Coffee intake, cups/day, No. (%)				
Characteristic	0	1	2	3	≥ 4		0	1	2	3	≥ 4
Total	23,859 (20.1)	9928 (8.4)	16,822 (14.2)	17,006 (14.3)	51,257 (43.1)		36,626 (30.8)	22,298 (18.8)	20,933 (17.6)	13,874 (11.7)	25,145 (21.2)
Age, median (IQR), year	58 (12)	58 (14)	59 (12)	60 (11)	60 (11)		58 (13)	60 (11)	60 (12)	60 (11)	60 (11)
Female, n (%)	12,411 (52.0)	4740 (47.7)	8250 (49.0)	8862 (52.1)	25,792 (50.3)		19,950 (54.5)	11,789 (52.9)	10,370 (49.5)	6691 (48.2)	11,255 (44.8)
White ethnicity, n (%)	22,804 (95.6)	8740 (88.0)	14,651 (86.9)	15,341 (90.2)	48,829 (95.3)		32,062 (87.5)	20,442 (91.7)	19,860 (94.9)	13,379 (96.4)	24,586 (97.8)
Education, n (%)											
Higher	6141 (25.7)	3045 (30.7)	4612 (27.4)	4420 (26.0)	11,395 (22.2)		7945 (21.7)	5730 (25.7)	5687 (27.2)	3901 (28.1)	6350 (25.3)
Middle	8086 (33.8)	3319 (33.4)	5433 (32.3)	5338 (31.4)	15,481 (30.2)		11,243 (30.7)	6911 (31.0)	6843 (32.7)	4558 (32.9)	8084 (32.1)
Lower	3227 (13.5)	1217 (12.3)	2134 (12.7)	2120 (12.5)	7123 (13.9)		5045 (13.8)	2843 (12.8)	2521 (12.0)	1747 (12.6)	3665 (14.6)
Vocational	1388 (5.8)	497 (5.0)	938 (5.6)	992 (5.8)	3023 (5.9)		2023 (5.5)	1377 (6.2)	1200 (5.7)	829 (6.0)	1409 (5.6)
Other	5035 (21.1)	1850 (18.6)	3705 (22.0)	4136 (24.3)	14,235 (27.8)		10,370 (28.3)	5433 (24.4)	4682 (22.4)	2839 (20.5)	5637 (22.4)
Socioeconomic status, n (%)											
Higher	4506 (18.9)	1988 (20.0)	3456 (20.5)	3590 (21.1)	10,248 (20.0)		6221 (17.0)	4659 (20.9)	4568 (21.8)	3137 (22.6)	5023 (20.7)
Intermediate	14,334 (60.1)	5946 (59.9)	9953 (59.2)	10,212 (60.0)	30,866 (60.2)		21,542 (58.8)	13,411 (60.2)	12,692 (60.6)	8450 (60.9)	15,216 (60.5)
Lower	5019 (21.0)	1994 (20.1)	3413 (20.3)	3024 (18.8)	10,143 (19.8)		8863 (24.2)	4224 (18.9)	3673 (17.5)	2287 (16.5)	4726 (18.8)
BMI, n (%)											
< 25 kg/m^2^	1014 (4.2)	542 (5.5)	1000 (5.9)	1006 (5.9)	2812 (5.5)		2280 (6.2)	1376 (6.2)	1118 (5.3)	616 (4.4)	984 (3.9)
25-29.9 kg/m^2^	7715 (32.3)	3683 (37.1)	6328 (37.6)	6543 (38.5)	18,961 (37.0)		13,241 (36.2)	8520 (38.2)	7881 (37.6)	5084 (36.6)	8504 (33.8)
≥ 30 kg/m^2^	15,130 (63.4)	5703 (57.4)	9494 (56.4)	9457 (55.6)	29,484 (57.5)		21,105 (57.6)	12,398 (55.6)	11,934 (57.0)	8174 (58.9)	15,657 (62.3)
Smoking status, n (%)											
Never	11,474 (48.1)	5035 (50.7)	8847 (52.6)	9127 (53.7)	24,999 (48.8)		20,069 (54.8)	11,738 (52.7)	10,443 (49.9)	6814 (49.1)	10,418(41.4)
Previous	9042 (37.9)	3764 (37.9)	6347 (37.7)	6377 (37.5)	20,030 (39.1)		12,842 (35.1)	8734 (39.2)	8401 (40.1)	5527 (39.8)	10,056 (40.0)
Current	3343 (14.0)	1129 (11.4)	1628 (9.7)	1502 (8.8)	6228 (12.2)		3715 (10.1)	1822 (8.2)	2089 (10.0)	1533 (11.0)	4671 (18.6)
Alcohol consumption, n (%)											
Low	7090 (29.7)	2467 (24.8)	4383 (26.1)	4417 (26.0)	14,395 (28.1)		13,498 (36.9)	5691 (25.5)	4503 (21.5)	2946 (21.2)	6114 (24.3)
Moderate	8787 (36.8)	3665 (36.9)	6170 (36.7)	6665 (39.2)	21,017 (41.0)		13,497 (36.9)	8957 (40.2)	8400 (40.1)	5405 (39.0)	10,045 (39.0)
High	7982 (33.5)	3796 (38.2)	6269 (37.3)	5924 (34.8)	15,845 (30.9)		9631 (26.3)	7646 (34.3)	8030 (38.4)	5523 (39.8)	8986 (35.7)
Physical activity, n (%)											
Low	7644 (32.0)	3042 (30.6)	4778 (28.4)	4645 (27.3)	14,151 (27.6)		10,665 (29.1)	5945 (26.7)	5717 (27.3)	4070 (29.3)	7863 (31.3)
Moderate	12,867 (53.9)	5434 (54.7)	9525 (56.6)	9888 (58.1)	28,938 (56.5)		20,384 (55.7)	12,838 (57.6)	11,950 (57.1)	7831 (56.4)	13,649 (54.3)
High	3348 (14.0)	1452 (14.6)	2519 (15.0)	2473 (14.5)	8168 (15.9)		5577 (15.2)	3511 (15.7)	3266 (15.6)	1973 (14.2)	3633 (14.4)
Healthy diet, n (%)	10,199 (42.7)	4648 (46.8)	7841 (46.6)	7998 (47.0)	22,798 (44.5)		16,074 (43.9)	10,810 (48.5)	9929 (47.4)	6225 (44.9)	10,446 (41.5)
Medication, n (%)											
Cholesterol-lowering drug, n (%)	7527 (31.5)	3096 (31.2)	5237 (31.1)	5278 (31.0)	15,846 (30.9)		11,217 (30.6)	7098 (31.8)	6499 (31.0)	4365 (31.5)	7805 (31.0)
Antihypertensive drug, n (%)	8553 (35.8)	3672 (37.0)	6288 (37.4)	6495 (38.2)	19,281 (37.6)		14,087 (38.5)	8628 (38.7)	7779 (37.2)	5136 (37.0)	8659 (34.4)
Insulin, n (%)	856 (3.6)	287 (2.9)	524 (3.1)	504 (3.0)	1549 (3.0)		1108 (3.0)	636 (2.9)	609 (2.9)	456 (2.9)	911 (3.3)
Depression, n (%)	2562 (10.7)	864 (8.7)	1462 (8.7)	1543 (9.1)	5490 (10.7)		3951 (10.8)	2097 (9.4)	1881 (9.0)	1341 (9.7)	2651 (10.5)

Percentages may not add up to 100% due to rounding. Binary variables display only one of the options

Abbreviations: SD, standard deviation; IQR, interquartile range;

All *p*-value < 0.05, except for diet in tea (*p*-value = 0.1) and coffee (*p-*value = 0.137), and depression in coffee (*p*-value = 0.107)

### Nonlinear association of tea and coffee consumption with mortality

**Fig. 1:**
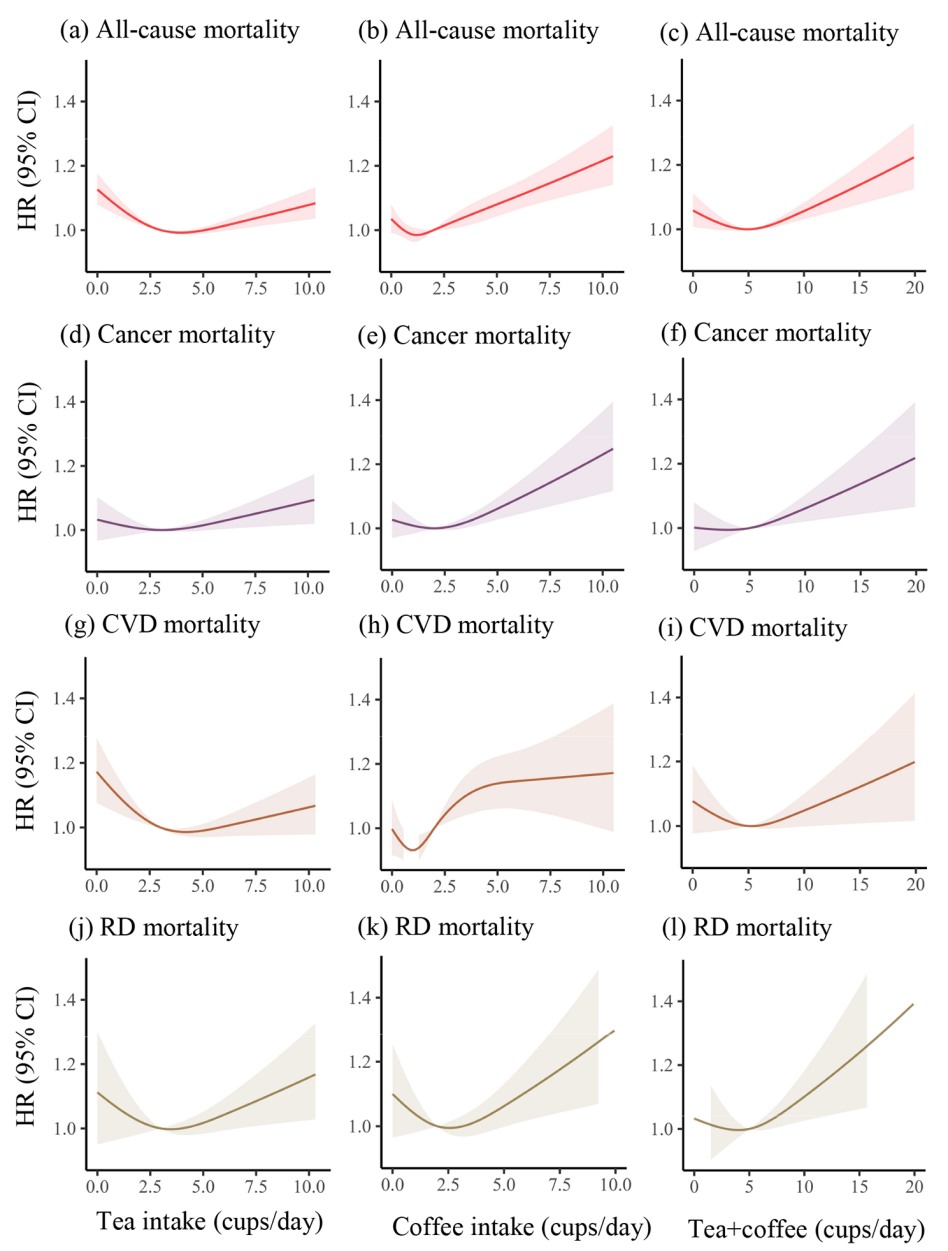
Dose-response associations of individual and combined tea and coffee consumption with all-cause and cause-specific mortality. The dose-response associations were examined in the Cox proportional hazard regression models based on restricted cubic splines with 3 knots, models were adjusted for age, sex, ethnicity, education, socioeconomic status, smoking status, alcohol consumption frequency, physical activity frequency, BMI, diet, cholesterol-lowering medication, BP medication, insulin, depression, and tea or coffee intake in coffee or tea analysis. The shaded area represents the 95% CI for the dose-response curve. Abbreviations: HR, Hazard ratio; CI, confidence interval; CVD, cardiovascular diseases; RD, respiratory disease. All *p* for nonlinearity < 0.001

During a median follow-up of 13.87 years (1,587,069 total person-years), 13,666 all-cause mortalities were recorded, including 5913 cancer deaths, 3362 CVD deaths, and 994 RD deaths. The dose-response relationships revealed a U-shaped nonlinear association between tea and coffee intake and all-cause, cancer, CVD, and RD mortality (all *p*-nonlinear < 0.001), and the consumption of approximately one cup of coffee or two cups of tea per day presented the lowest all-cause mortality risk. The combined intake of tea and coffee showed a U-shaped nonlinear relationship with all-cause, CVD, and RD mortality and a J-shaped nonlinear relationship with cancer death (all *p*-nonlinear < 0.001); the combined consumption of tea and coffee around 4–5 cups/day was linked to lower levels of mortality risk (Fig. 1).

### Association of separate tea and coffee consumption with mortality

**Fig. 2:**
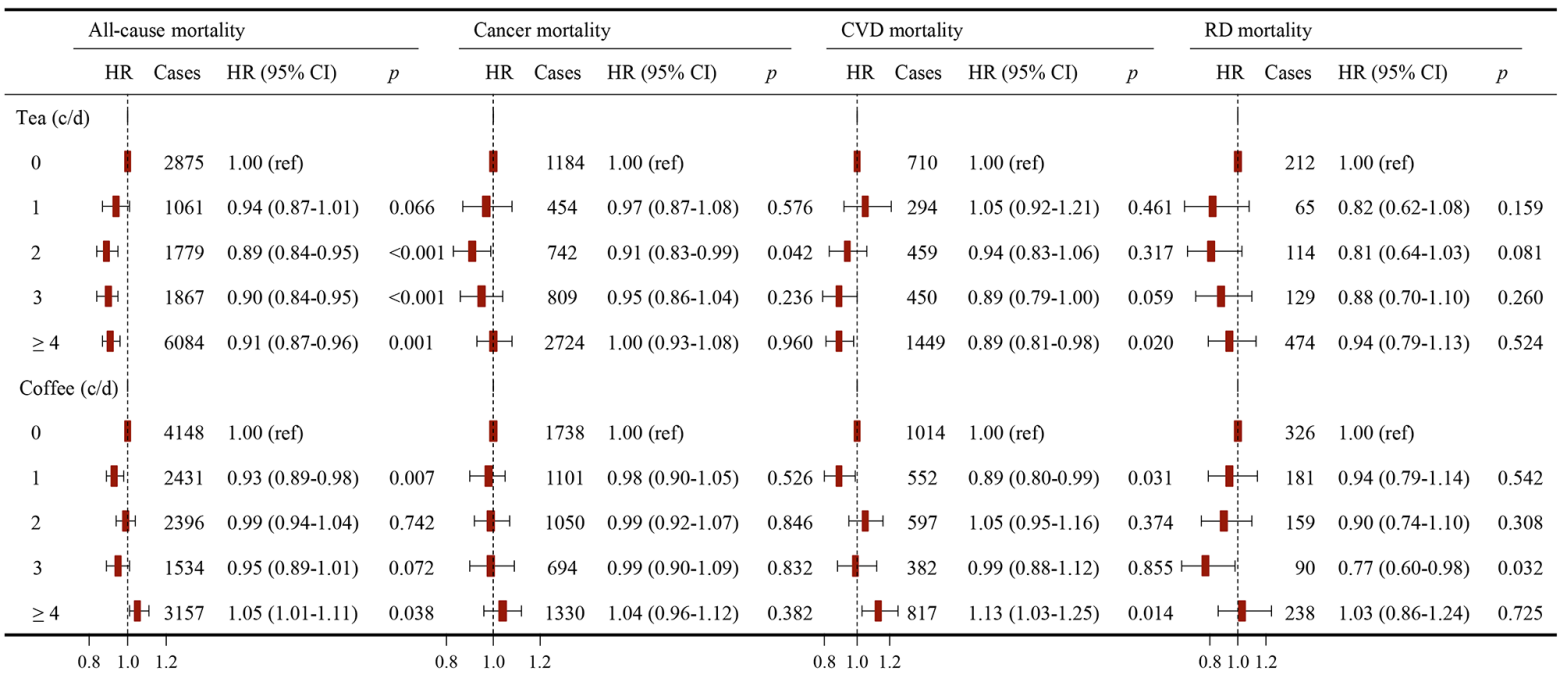
HRs (95% CIs) for all-cause and cause-specific mortality according to individual tea and coffee consumption. Models were adjusted for age, sex, ethnicity, education, socioeconomic status, smoking status, alcohol consumption frequency, physical activity frequency, BMI, diet, cholesterol-lowering medication, BP medication, insulin, depression, and tea or coffee intake in coffee or tea analysis. Abbreviations: HR, Hazard ratio; CI, confidence interval; CVD, cardiovascular diseases; RD, respiratory disease; c/d, cups/day

To quantify the magnitude of tea and coffee consumption with mortality, we categorised tea and coffee intake as follows:0, 1, 2, 3, ≥ 4 cups/day. In multivariable-adjusted Cox model three, compared with non-tea drinkers, those who drank tea 2, 3, or ≥ 4 cups/day were inversely related to all-cause mortality risk, with HRs (95% CI) of 0.89 (0.84–0.95), 0.90 (0.84–0.95), and 0.91 (0.87–0.96), respectively. Meanwhile, drinking 2 cups or ≥ 4 cups of tea per day was linked to a decreased risk of cancer mortality (HR 0.91, 95% CI 0.83–0.99) or CVD mortality (HR 0.89, 95% CI 0.81–0.98). Additionally, we observed the protective links between coffee intake of 1 cup/day and all-cause and CVD mortality, and the respective HRs (95% CI) for 1 cup/day vs. non-coffer consumers were 0.93 (0.89–0.98) and 0.89 (0.80–0.99). Coffee intake of 3 cups/day also had a protective effect on RD mortality (HR 0.77, 95% CI 0.60–0.98). However, coffee intake of ≥ 4 cups/day displayed a higher risk of all-cause (HR 1.05, 95% CI 1.01–1.11) and CVD mortality (HR 1.13, 95% CI 1.03–1.25) compared with non-coffee drinkers (Fig. 2). The results of Models 1 and 2 were consistent with this finding (Supplementary Table S3).

### Association of combined tea and coffee consumption with mortality

**Fig. 3:**
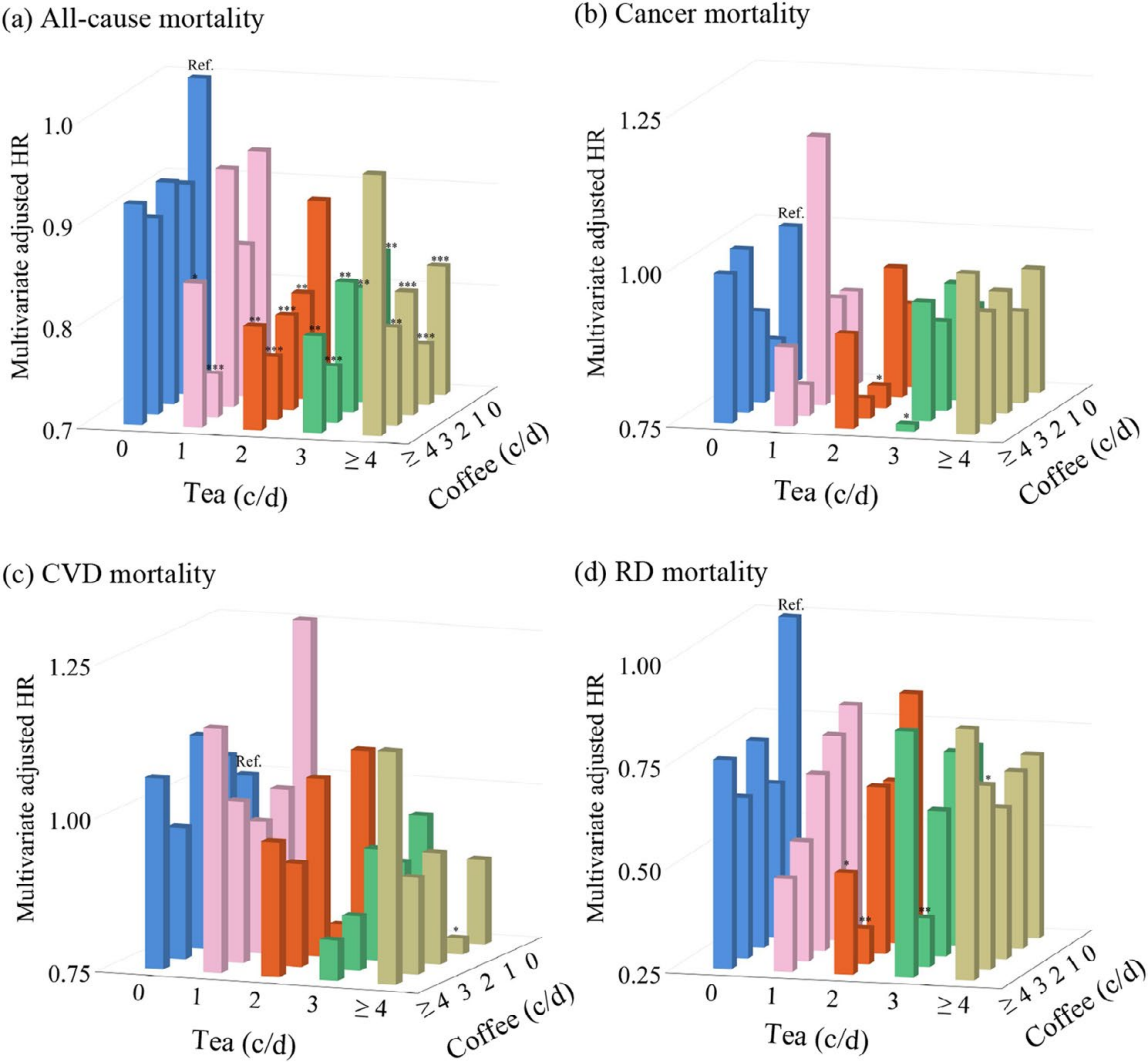
Combined consumption of tea and coffee with all-cause and cause-specific mortality. Models were adjusted for age, sex, ethnicity, education, socioeconomic status, smoking status, alcohol consumption frequency, physical activity frequency, BMI, diet, cholesterol-lowering medication, BP medication, insulin, depression, and tea or coffee intake in coffee or tea analysis. Individuals who did not consume tea and coffee were considered a reference group (Ref.). Abbreviations: HR, Hazard ratio; CVD, cardiovascular diseases; RD, respiratory disease; c/d, cups/day; *, *p* < 0.05; **, *p* < 0.01; ***, *p* < 0.001

We further explored the joint effects of combined tea and coffee consumption on mortality. As shown in Fig. 3 and Supplementary Table S4, compared to individuals who did not consume tea and coffee, tea and coffee drinkers revealed a reduced risk of all-cause mortality (HR < 1). Specifically, individuals who consumed 2 cups of tea plus 2 cups of coffee daily, or 3 cups of tea plus ≥ 4 cups of coffee daily, showed 21% (HR 0.79, 95% CI 0.63–0.98) or 24% (HR 0.76, 95% CI 0.58–0.99) lower risk of cancer mortality, respectively. Moreover, participants who drank both 4 cups of tea and a cup of coffee per day experienced a 22% lower risk of death from CVD (HR 0.78, 95% CI 0.61–0.99). Additionally, the combined effect of tea and coffee consumption decreased the risk of death from RD (HR < 1).

### Subgroup analyses and sensitivity analyses

**Fig. 4:**
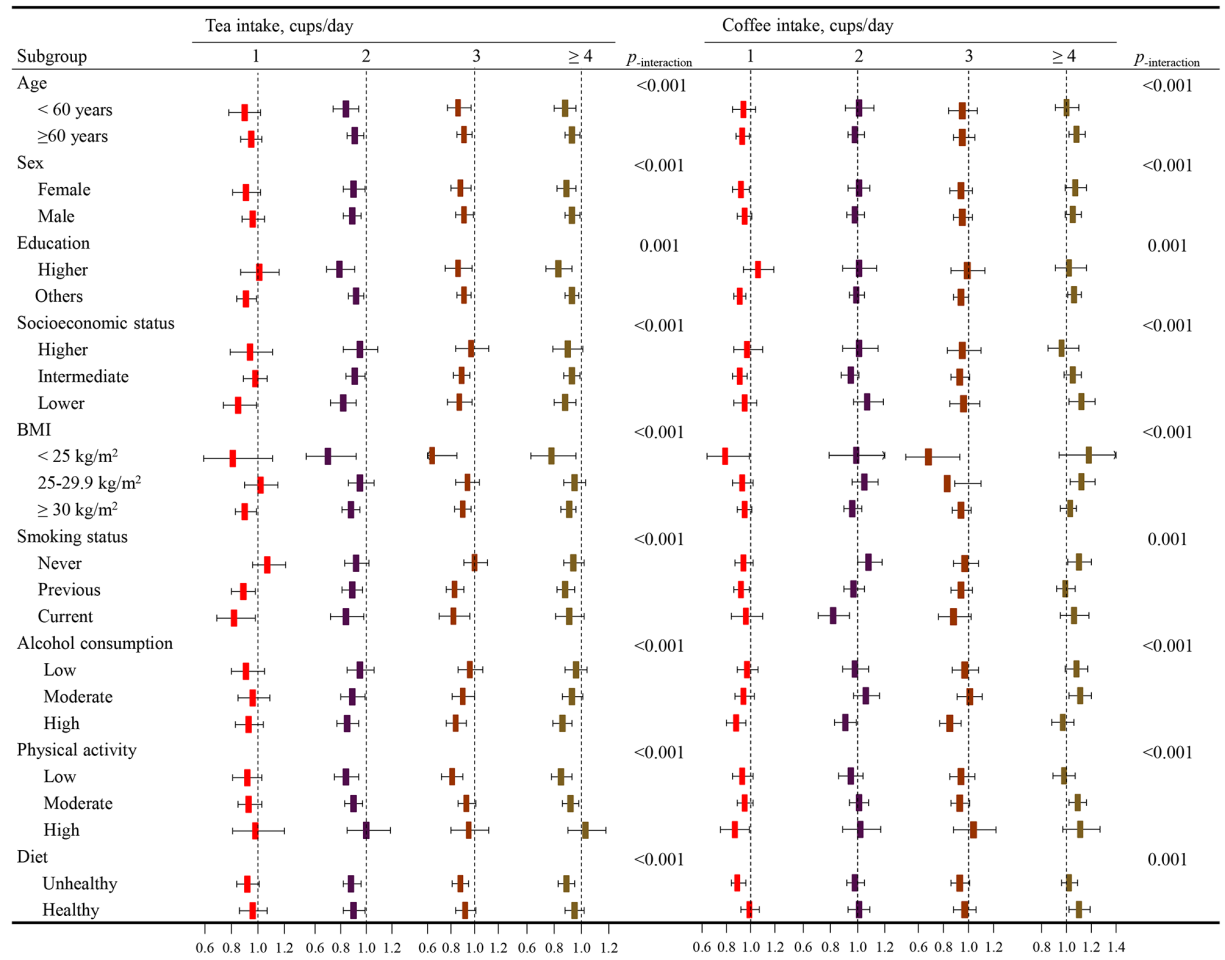
HRs (95% CI) of separate tea and coffee consumption for all-cause mortality in subgroups. Models were adjusted for age, sex, ethnicity, education, socioeconomic status, smoking status, alcohol consumption frequency, physical activity frequency, BMI, diet, cholesterol-lowering medication, BP medication, insulin, depression, and tea or coffee intake in coffee or tea analysis. Non-tea drinkers or non-coffee drinkers were considered the reference group. *p*_*−* interaction_: the *p* for interaction was estimated by including an interaction term beta of the tea/coffee-baseline characteristics in fully adjusted Cox models

The results of the subgroup analyses supported the protective effect of one cup of coffee daily against the risk of death, and consuming coffee ≥ 4 cups/day revealed a significant increase in the risk of death (Fig. 4 and Supplementary Table S5). In addition, the links between tea intake and death were more pronounced in those who were < 60 years old, female, highly educated, economically disadvantaged, low BMI, current smokers, regular alcohol drinkers, less physically active, or had an unhealthy diet, etc. (*p* for interaction < 0.01). Moreover, sensitivity analyses showed that tea intake and moderate coffee consumption were related to a decreased risk of death when unadjusted for depression at recruitment or after removing patients with cancer and CVD at baseline (Supplementary Table S6-S7).

## Discussion

In this prospective study of 118,872 patients with MetS, we found the U-shaped nonlinear associations between separate tea and coffee intake and mortality (all *p*-nonlinear < 0.001). Compared with non-tea consumers, tea consumers appear to have a decreased risk of all-cause, cancer, CVD, and RD mortality, whereas moderate coffee consumption (1 or 3 cups/day) demonstrates a lower all-cause, CVD, and RD mortality risk. However, excessive coffee consumption (≥ 4 cups/day) increases the risk of all-cause and CVD mortality. Notably, the simultaneous consumption of tea and coffee was inversely associated with mortality risk.

Numerous studies have linked tea consumption to a decreased risk of death in the general population. A cohort of over 100,000 general individuals from China found that habitual tea drinkers had 20% and 22% lower risks of CVD and all-cause mortality, respectively [20]. A similar pattern of effect was detected in two cohort studies of approximately half a million Britons [14, 21]. Notably, a prospective cohort study of 15,486 participants with type 2 diabetes mellitus (DM) in the United States also documented a protective effect of tea consumption on mortality [22]. Despite the growing focus on the association between tea consumption and mortality, evidence from patients with MetS has been poorly examined. Although this study found a U-shaped dose-response association between tea intake and death in patients with MetS, tea consumption ≥ 4 cups/day still showed a protective effect against death compared to non-tea consumption. This implies that tea consumption positively impacts overall health and longevity, complementing previous results in populations with MetS.

Bioactive compounds in tea, such as catechins and flavonoids, have been linked to antioxidant, anti-inflammatory, antiviral, and anti-carcinogenic properties, which may contribute to the observed protective effects against mortality [23–26]. The phytochemicals present in tea, especially polyphenols, have been suggested for consideration in the prevention and treatment of respiratory-related diseases through their ability to improve immunity and modulate the gut-lung axis [27]. They have also been demonstrated to possess anti-cancer properties by inhibiting tumour cell proliferation and inducing apoptosis [28]. Furthermore, the antihypertensive [29], lipid-lowering [30], hypoglycemic [31], anti-atherosclerotic [32], and anti-arrhythmic properties of tea may lower the risk of CVD-related death [33].

Currently, the observed U-shaped or J-shaped relationship between coffee consumption and mortality in different populations remains controversial [34, 35]. Several studies have reported protective associations between coffee consumption and death [22, 36, 37]. Notably, a Danish observational study discovered a U-shaped association between coffee intake, CVD, and all-cause mortality, which is consistent with the findings of this study in a population with MetS [34]. Moreover, a recent analysis by Chen et al. proposed a J-shaped association between coffee intake and all-cause mortality risk but presented a U-shaped relationship between coffee intake and RD death risk; compared with non-coffee drinkers, consuming coffee 1–2 cups/day presented a 33% lower risk of death from RD, and drinking coffee ≥ 5 cups/day increased the risk by 61% [14]. These inconsistent findings may be attributed to variations in racial backgrounds and underlying health conditions such as DM and MetS. Our findings suggest that null or excessive coffee intake could potentially increase the risk of death, emphasising the importance of moderate coffee consumption in individuals with MetS.

The U-shaped association between coffee consumption and all-cause and CVD mortality may be linked to multiple mechanisms. The first possible mechanism is the presence of bioactive compounds in coffee, such as polyphenols and antioxidants [38–40]. These compounds have demonstrated anti-inflammatory and antioxidant properties that may help reduce the risk of CVD [41]. They can also protect against oxidative stress and DNA damage, the underlying factors in various chronic diseases [42]. Coffee contains caffeine, which has been linked to several health benefits [43]. Caffeine improves the release of nitric oxide from endothelial cells, dilates blood vessels, and enhances antioxidant capacity [44]. These effects may indirectly contribute to reduced risks of CVD and death. Additionally, coffee consumption has been linked to a reduced likelihood of type 2 DM, which is a significant predisposing factor for CVD [45]. Compounds in coffee, such as chlorogenic acid, may improve insulin sensitivity and glucose metabolism, leading to a decreased risk of DM and subsequent CVD complications [46]. However, excessive coffee consumption may promote all-cause and CVD mortality via different mechanisms. High caffeine consumption can increase blood pressure and heart rate, potentially increasing cardiovascular stress and CVD [47]. Excessive caffeine consumption can also disrupt sleep patterns and lead to sleep deprivation, which has been linked to various health problems including CVD [48].

Interestingly, although excessive coffee consumption may increase the likelihood of mortality in patients with MetS, the simultaneous consumption of coffee and tea can potentially lower this risk. This indicates a potential synergistic effect between tea and coffee consumption in reducing mortality risk. To date, few studies have explored the combined effect of coffee and tea on the risk of death; only two studies have investigated the additive effects of tea and coffee on reducing the risk of death [13, 14], consistent with the findings of this study in populations with MetS. The underlying mechanisms may involve the combined action of bioactive compounds and improvements in insulin sensitivity, glucose metabolism, blood pressure regulation, and other physiological pathways [49, 50]. Further research is required to fully understand the specific mechanisms and optimal dosage of coffee and tea consumption to reduce mortality risk among patients with MetS.

The study’s main strengths lie in its extensive sample size and specific focus on the populations with MetS. By examining the relationship between tea and coffee consumption and mortality in patients with MetS, this research aims to enhance the current scientific literature and provide evidence-based information for the development of preventive strategies and interventions. Ultimately, these findings have significant implications for public health policies and the promotion of healthy dietary habits among individuals with MetS.

This study has a few limitations. First, the observed associations were based on self-reported dietary intake, which may have been subject to a recall bias. Second, residual confounding factors not considered in the analysis may have influenced the results. Third, while focusing on patients with MetS is advantageous, it may limit the generalisability of the findings, which may not apply to other populations with different characteristics. Finally, because the study was purely observational, no causality could be established.

## Conclusions

This study provides evidence of a U-shaped nonlinear relationship between separate tea and coffee consumption and all-cause and cause-specific mortality. The risk of death was at a low level when consuming 2–4 cups/day of tea or 1–3 cups/day of coffee, and the excessive coffee consumption (≥ 4 cups/day) increases the risk of all-cause and CVD mortality. Notably, simultaneous intake of tea and coffee presented a protective effect against mortality risk. In conclusion, daily tea intake and moderate coffee consumption can be suggested as components of healthy lifestyles to optimise health benefits and diminish the likelihood of mortality. These findings emphasise the importance of incorporating tea and moderate coffee consumption into the diets of individuals with MetS to improve their health.

### Electronic supplementary material

Below is the link to the electronic supplementary material.

Supplementary Material 1. **Supplementary Tables**. **Table S1**. Coding of outcomes. **Table S2.** Baseline characteristics by combined tea and coffee intake. **Table S3.** HR (95% CI) of separate tea and coffee consumption with mortality. **Table S4.** HR (95% CI) of combined tea and coffee consumption with mortality. **Table S5.** HR (95% CI) of separate tea and coffee consumption with all-cause mortality by baseline characteristics. **Table S6.** HR (95% CI) of separate tea and coffee consumption with all-cause and cause-specific mortality when unadjusted for depression at recruitment. **Table S7.** HR (95% CI) of separate tea and coffee consumption with all-cause and cause-specific mortality after exclusion of individuals with CVD and cancer at recruitment (n = 98,102). **Supplementary Figure**. **Supplementary Methods**. **S1**. Assessment of covariates. **S2.** Assessment of missing values.

## Data Availability

The UK Biobank datasets are openly available by submitting a data request proposal from https://www.ukbiobank.ac.uk/ (We accessed on 10 April 2023). We are authorized to access the database through the Access Management System (AMS) (Application number: 78,563).

## Abbreviations

MetS: metabolic syndrome
HR: Hazard ratio
CI: confidence interval
CVD: cardiovascular diseases
RD: respiratory disease
BP: blood pressure
FG: fasting glucose
HDLC: high-density lipoprotein cholesterol
TG: triglycerides
NHS: National Health Service
BMI: body mass index
SD: standard deviation
IQR: interquartile range
RCS: restricted cubic splines
c/d: cups/day
DM: diabetes mellitus

## References

[1] Alberti KG, Eckel RH, Grundy SM, Zimmet PZ, Cleeman JI, Donato KA, Fruchart JC, James WP, Loria CM, Smith SC (2009). Harmonizing the metabolic syndrome: a joint interim statement of the International Diabetes Federation Task Force on Epidemiology and Prevention; National Heart, Lung, and Blood Institute; American Heart Association; World Heart Federation; International Atherosclerosis Society; and International Association for the Study of Obesity. Circulation.

[2] Saklayen MG (2018). The global epidemic of the metabolic syndrome. Curr Hypertens Rep.

[3] Peiris CL, van Namen M, O’Donoghue G (2021). Education-based, lifestyle intervention programs with unsupervised exercise improve outcomes in adults with metabolic syndrome. A systematic review and meta-analysis. Rev Endocr Metab Disord.

[4] Tran TT, Gunathilake M, Lee J, Kim J (2022). Association between metabolic syndrome and its components and incident Colorectal cancer in a prospective cohort study. Cancer.

[5] Wu E, Ni JT, Zhu ZH, Xu HQ, Tao L, Xie T (2022). Association of a healthy lifestyle with All-Cause, cause-Specific Mortality and Incident Cancer among individuals with metabolic syndrome: a prospective cohort study in UK Biobank. Int J Environ Res Public Health.

[6] Boudreau DM, Malone DC, Raebel MA, Fishman PA, Nichols GA, Feldstein AC, Boscoe AN, Ben-Joseph RH, Magid DJ, Okamoto LJ (2009). Health care utilization and costs by metabolic syndrome risk factors. Metab Syndr Relat Disord.

[7] Pérez-Martínez P, Mikhailidis DP, Athyros VG, Bullo M, Couture P, Covas MI, de Koning L, Delgado-Lista J, Díaz-López A, Drevon CA (2017). Lifestyle recommendations for the prevention and management of metabolic syndrome: an international panel recommendation. Nutr Rev.

[8] Baspinar B, Eskici G, Ozcelik AO (2017). How coffee affects metabolic syndrome and its components. Food Funct.

[9] Yang CS, Zhang J, Zhang L, Huang J, Wang Y (2016). Mechanisms of body weight reduction and metabolic syndrome alleviation by tea. Mol Nutr Food Res.

[10] Van Dam RM, Hu FB, Willett WC (2020). Coffee, Caffeine, and Health. N Engl J Med.

[11] Sirotkin AV, Kolesárová A (2021). The anti-obesity and health-promoting effects of tea and coffee. Physiol Res.

[12] Esmaeelpanah E, Razavi BM, Hosseinzadeh H (2021). Green tea and metabolic syndrome: a 10-year research update review. Iran J Basic Med Sci.

[13] Komorita Y, Iwase M, Fujii H, Ohkuma T, Ide H, Jodai-Kitamura T, Yoshinari M, Oku Y, Higashi T, Nakamura U (2020). Additive effects of green tea and coffee on all-cause mortality in patients with type 2 Diabetes Mellitus: the Fukuoka Diabetes Registry. BMJ open Diabetes Research & care.

[14] Chen Y, Zhang Y, Zhang M, Yang H, Wang Y (2022). Consumption of coffee and tea with all-cause and cause-specific mortality: a prospective cohort study. BMC Med.

[15] Sudlow C, Gallacher J, Allen N, Beral V, Burton P, Danesh J, Downey P, Elliott P, Green J, Landray M (2015). UK biobank: an open access resource for identifying the causes of a wide range of complex Diseases of middle and old age. PLoS Med.

[16] Wu E, Guo JP, Wang K, Xu HQ, Xie T, Tao L, Ni JT (2023). Association of serum 25-hydroxyvitamin D with the incidence of 16 cancers, cancer mortality, and all-cause mortality among individuals with metabolic syndrome: a prospective cohort study. Eur J Nutr.

[17] Eastwood SV, Mathur R, Atkinson M, Brophy S, Sudlow C, Flaig R, de Lusignan S, Allen N, Chaturvedi N (2016). Algorithms for the capture and Adjudication of Prevalent and Incident Diabetes in UK Biobank. PLoS ONE.

[18] Ong JS, Law MH, An J, Han X, Gharahkhani P, Whiteman DC, Neale RE, MacGregor S (2019). Association between coffee consumption and overall risk of being diagnosed with or dying from cancer among > 300 000 UK Biobank participants in a large-scale mendelian randomization study. Int J Epidemiol.

[19] Han H, Cao Y, Feng C, Zheng Y, Dhana K, Zhu S, Shang C, Yuan C, Zong G (2022). Association of a healthy lifestyle with all-cause and cause-specific mortality among individuals with type 2 Diabetes: a prospective study in UK Biobank. Diabetes Care.

[20] Wang X, Liu F, Li J, Yang X, Chen J, Cao J, Wu X, Lu X, Huang J, Li Y (2020). Tea consumption and the risk of atherosclerotic Cardiovascular Disease and all-cause mortality: the China-PAR project. Eur J Prev Cardiol.

[21] Inoue-Choi M, Ramirez Y, Cornelis MC, Berrington de González A, Freedman ND, Loftfield E (2022). Tea consumption and all-cause and cause-specific mortality in the UK Biobank: a prospective cohort study. Ann Intern Med.

[22] Ma L, Hu Y, Alperet DJ, Liu G, Malik V, Manson JE, Rimm EB, Hu FB, Sun Q (2023). Beverage consumption and mortality among adults with type 2 Diabetes: prospective cohort study. BMJ.

[23] Chlorinated. drinking-water; chlorination by-products; some other halogenated compounds; cobalt and cobalt compounds. International Agency for Research on Cancer (IARC) Working Group, Lyon, 12–19 June 1990. *IARC Monogr Eval Carcinog Risks Hum* 1991, 52:1-544. PMC7681469 1683674

[24] Raman G, Avendano EE, Chen S, Wang J, Matson J, Gayer B, Novotny JA, Cassidy A (2019). Dietary intakes of flavan-3-ols and cardiometabolic health: systematic review and meta-analysis of randomized trials and prospective cohort studies. Am J Clin Nutr.

[25] Paiva L, Lima E, Motta M, Marcone M, Baptista J (2022). Investigation of the Azorean Camellia sinensis Processing conditions to maximize the Theaflavin 3,3’-di-O-Gallate content as a potential antiviral compound. Antioxid (Basel Switzerland).

[26] Eggers M, Jungke P, Wolkinger V, Bauer R, Kessler U, Frank B (2022). Antiviral activity of plant juices and green tea against SARS-CoV-2 and Influenza virus. Phytother Res.

[27] Xu L, Ho CT, Liu Y, Wu Z, Zhang X (2022). Potential application of tea polyphenols to the Prevention of COVID-19 Infection: based on the gut-lung Axis. Front Nutr.

[28] Parish M, Massoud G, Hazimeh D, Segars J, Islam MS (2023). Green Tea in Reproductive cancers: could Treatment be as simple?. Cancers (Basel).

[29] Mahdavi-Roshan M, Salari A, Ghorbani Z, Ashouri A (2020). The effects of regular consumption of green or black tea beverage on blood pressure in those with elevated blood pressure or Hypertension: a systematic review and meta-analysis. Complement Ther Med.

[30] Xu R, Yang K, Li S, Dai M, Chen G (2020). Effect of green tea consumption on blood lipids: a systematic review and meta-analysis of randomized controlled trials. Nutr J.

[31] Neyestani TR, Nikooyeh B (2022). A comprehensive overview on the effects of green tea on anthropometric measures, blood pressure, glycemic and lipidemic status: an umbrella review and meta meta-analysis study. Nutr Metabolism Cardiovasc Diseases: NMCD.

[32] Dludla PV, Nkambule BB, Mazibuko-Mbeje SE, Nyambuya TM, Orlando P, Silvestri S, Marcheggiani F, Cirilli I, Ziqubu K, Ndevahoma F (2021). Tea consumption and its effects on primary and secondary prevention of coronary artery Disease: qualitative synthesis of evidence from randomized controlled trials. Clin Nutr ESPEN.

[33] Voskoboinik A, Kalman JM, Kistler PM (2018). Caffeine and arrhythmias: time to grind the data. JACC Clin Electrophysiol.

[34] Nordestgaard AT, Nordestgaard BG (2016). Coffee intake, Cardiovascular Disease and all-cause mortality: observational and mendelian randomization analyses in 95 000-223 000 individuals. Int J Epidemiol.

[35] Ruggiero E, Di Castelnuovo A, Costanzo S, Persichillo M, De Curtis A, Cerletti C, Donati MB, de Gaetano G, Iacoviello L, Bonaccio M (2021). Daily Coffee drinking is Associated with Lower risks of Cardiovascular and total mortality in a General Italian Population: results from the Moli-Sani Study. J Nutr.

[36] Loftfield E, Cornelis MC, Caporaso N, Yu K, Sinha R, Freedman N (2018). Association of Coffee drinking with mortality by genetic variation in Caffeine Metabolism: findings from the UK Biobank. JAMA Intern Med.

[37] Kim SA, Tan LJ, Shin S (2021). Coffee Consumption and the risk of all-cause and cause-specific mortality in the Korean Population. J Acad Nutr Dietetics.

[38] Ősz BE, Jîtcă G, Ștefănescu RE, Pușcaș A, Tero-Vescan A, Vari CE (2022). Caffeine and its antioxidant Properties-It is all about dose and source. Int J Mol Sci.

[39] De Melo Pereira GV, de Carvalho Neto DP, Magalhães Júnior AI, do Prado FG, Pagnoncelli MGB, Karp SG, Soccol CR (2020). Chemical composition and health properties of coffee and coffee by-products. Adv Food Nutr Res.

[40] Kolb H, Kempf K, Martin S. Health effects of Coffee: mechanism unraveled? *Nutrients* 2020, 12(6):1842. 10.3390/nu12061842. 10.3390/nu12061842 PMC7353358 32575704

[41] María Mérida D, Vitelli-Storelli F, Moreno-Franco B, Rodríguez-Ayala M, López-García E, Banegas JR, Rodríguez-Artalejo F, Guallar-Castillón P (2023). Polyphenol intake and mortality: a nationwide cohort study in the adult population of Spain. Clin Nutr.

[42] O’Keefe JH, Bhatti SK, Patil HR, DiNicolantonio JJ, Lucan SC, Lavie CJ (2013). Effects of habitual coffee consumption on cardiometabolic Disease, cardiovascular health, and all-cause mortality. J Am Coll Cardiol.

[43] Barcelos RP, Lima FD, Carvalho NR, Bresciani G, Royes LF (2020). Caffeine effects on systemic metabolism, oxidative-inflammatory pathways, and exercise performance. Nutr Res (New York NY).

[44] Voskoboinik A, Koh Y, Kistler PM (2019). Cardiovascular effects of caffeinated beverages. Trends Cardiovasc Med.

[45] Akash MS, Rehman K, Chen S (2014). Effects of coffee on type 2 Diabetes Mellitus. Nutr (Burbank Los Angeles Cty Calif).

[46] Pimpley V, Patil S, Srinivasan K, Desai N, Murthy PS (2020). The chemistry of chlorogenic acid from green coffee and its role in attenuation of obesity and Diabetes. Prep Biochem Biotechnol.

[47] Zhang Z, Hu G, Caballero B, Appel L, Chen L (2011). Habitual coffee consumption and risk of Hypertension: a systematic review and meta-analysis of prospective observational studies. Am J Clin Nutr.

[48] Clark I, Landolt HP (2017). Coffee, caffeine, and sleep: a systematic review of epidemiological studies and randomized controlled trials. Sleep Med Rev.

[49] Tang J, Zheng JS, Fang L, Jin Y, Cai W, Li D (2015). Tea consumption and mortality of all cancers, CVD and all causes: a meta-analysis of eighteen prospective cohort studies. Br J Nutr.

[50] Alperet DJ, Rebello SA, Khoo EY, Tay Z, Seah SS, Tai BC, Tai ES, Emady-Azar S, Chou CJ, Darimont C (2020). The effect of coffee consumption on insulin sensitivity and other biological risk factors for type 2 Diabetes: a randomized placebo-controlled trial. Am J Clin Nutr.

