# Supplementary material for: Association of tea and coffee consumption with the risk of all-cause and cause-specific mortality among individuals with metabolic syndrome: a prospective cohort study
Source: Diabetol Metab Syndr. 2023 Nov 23;15:241. doi: 10.1186/s13098-023-01222-7 (PMC10666405; doi:10.1186/s13098-023-01222-7)
Supplement: Supplementary file 1 — Supplementary Material 1. Supplementary Tables. Table S1. Coding of outcomes. Table S2. Baseline characteristics by combined tea and coffee intake. Table S3. HR (95% CI) of separate tea and coffee consumption with mortality. Table S4. HR (95% CI) of combined tea and coffee consumption with mortality. Table S5. HR (95% CI) of separate tea and coffee consumption with all-cause mortality by baseline characteristics. Table S6. HR (95% CI) of separate tea and coffee consumption with all-cause and cause-specific mortality when unadjusted for depression at recruitment. Table S7. HR (95% CI) of separate tea and coffee consumption with all-cause and cause-specific mortality after exclusion of individuals with CVD and cancer at recruitment (n = 98,102). Supplementary Figure. Supplementary Methods. S1. Assessment of covariates. S2. Assessment of missing values. [file 13098_2023_1222_MOESM1_ESM.pdf]

Association of tea and coffee consumption with the risk of all-cause and cause-specific mortality among individuals with metabolic syndrome: a prospective cohort study

E Wu et al.

Supplementary material content

Supplementary Tables **2**

Table S1. Coding of outcomes 2

Table S2. Baseline characteristics by combined tea and coffee intake 3

Table S3. HR (95% CI) of separate tea and coffee consumption with mortality 4

Table S4. HR (95% CI) of combined tea and coffee consumption with mortality 5

Table S5. HR (95% CI) of separate tea and coffee consumption with all-cause mortality by baseline characteristics 6

Table S6. HR (95% CI) of separate tea and coffee consumption with all-cause and cause-specific mortality when unadjusted for depression at recruitment 8

Table S7. HR (95% CI) of separate tea and coffee consumption with all-cause and cause-specific mortality after exclusion of individuals with CVD and cancer at recruitment (n = 98102) 9

Supplementary Figure **10**

Fig. S1 Cohort exclusions of the study participants 10

Supplementary Methods **11**

S1. Assessment of covariates 11

S2. Assessment of missing values 12

References **13**

Supplementary Tables

Table S1. Coding of outcomes

Cause of death	ICD-10
Cancer mortality	C00-C97
Cardiovascular diseases mortality	I00-I99
Respiratory disease	J09-J98

The cause-specific mortality was recorded using the 10th International Classifcation of Diseases (ICD-10) codes.

Table S2. Baseline characteristics by combined tea and coffee intake

Characteristic	Coffee and tea intake, No. (%)			*p*-value
	None	Only one	Both	
Total	3764 (5.2)	52957 (44.5)	62151 (52.3)	
Age, median (IQR), year	55 (13)	59 (12)	60 (11)	<0.001
Female, n (%)	2205 (58.6)	27951 (52.8)	29899 (48.1)	<0.001
White ethnicity, n (%)	3345 (88.9)	48176 (91.0)	58808 (94.6)	<0.001
Education, n (%)				<0.001
Higher	1010 (26.8)	12066 (22.8)	16537 (26.6)	
Middle	1327 (35.3)	16657 (31.5)	19655 (31.6)	
Lower	486 (12.9)	7300 (13.8)	8035 (12.9)	
Vocational	204 (5.4)	3003 (5.7)	3631 (5.8)	
Other	737 (19.6)	13931 (26.3)	14293 (23.)	
Socioeconomic status, n (%)				<0.001
Higher	633 (16.8)	9461 (17.9)	13694 (22.0)	
Intermediate	2159 (57.4)	31588 (59.6)	37594 (60.5)	
Lower	972 (25.8)	11938 (22.5)	10863 (17.5)	
BMI, n (%)				<0.001
<25 kg/m^2	197 (5.2)	2900 (5.5)	3277 (5.3)	
25-29.9 kg/m^2	1158 (30.8)	18640 (35.2)	23432 (37.7)	
≥30 kg/m^2	2409 (64.0)	31417 (59.3)	35442 (57.0)	
Smoking status				<0.001
Never	2454 (65.2)	26635 (50.3)	30393 (48.9)	
Previous	1018 (27.0)	19848 (37.5)	24694 (39.7)	
Current	292 (7.8)	6474 (12.2)	7064 (11.4)	
Alcohol consumption, n (%)				<0.001
Low	1743 (46.3)	17102 (32.3)	13907 (22.4)	
Moderate	1264 (33.6)	19756 (37.3)	25284 (40.7)	
High	757 (20.1)	16099 (30.4)	22960 (36.9)	
Physical activity, n (%)				<0.001
Low	1152 (30.6)	16005 (30.2)	17103 (27.5)	
Moderate	2041 (54.2)	29169 (55.1)	35442 (57.0)	
High	571 (15.2)	7783 (14.7)	9606 (15.5)	
Healthy diet, n (%)	1771 (47.1)	22731 (42.9)	28982 (46.6)	<0.001
Cholesterol-lowering drug, n (%)	950 (25.2)	16844 (31.8)	19190 (30.9)	<0.001
Antihypertensive drug, n (%)	1200 (31.9)	20240 (38.2)	22849 (36.8)	<0.001
Insulin, n (%)	122 (3.2)	1720 (3.2)	1878 (3.0)	0.027
Depression, n (%)	443 (11.8)	5627 (10.6)	5851 (9.4)	0.104

Table S3. HR (95% CI) of separate tea and coffee consumption with mortality

		All-cause mortality		Cancer mortality		CVD mortality		Respiratory mortality	
Exposure	Model	HR (95% CI)	*p*	HR (95% CI)	*p*	HR (95% CI)	*p*	HR (95% CI)	*p*
Tea (cups/d)	Molel 1								
	0	1.00 (ref)		1.00 (ref)		1.00 (ref)		1.00 (ref)	
	1	0.88 (0.82-0.94)	<0.001	0.93 (0.83-1.03)	0.164	0.96 (0.84-1.10)	0.584	0.75 (0.56-0.99)	0.039
	2	0.82 (0.77-0.87)	<0.001	0.85 (0.77-0.93)	<0.001	0.83 (0.74-0.93)	0.002	0.71 (0.56-0.89)	0.003
	3	0.81 (0.76-0.86)	<0.001	0.87 (0.79-0.95)	0.002	0.78 (0.69-0.88)	<0.001	0.75 (0.60-0.93)	0.009
	≥4	0.85 (0.81-0.89)	<0.001	0.94 (0.88-1.01)	0.090	0.81 (0.74-0.89)	<0.001	0.86 (0.73-1.01)	0.071
	Molel 2								
	0	1.00 (ref)		1.00 (ref)		1.00 (ref)		1.00 (ref)	
	1	0.92 (0.86-0.99)	0.018	0.96 (0.86-1.07)	0.408	1.03 (0.90-1.18)	0.713	0.80 (0.60-1.06)	0.119
	2	0.87 (0.82-0.92)	<0.001	0.89 (0.81-0.97)	0.010	0.90 (0.80-1.01)	0.081	0.78 (0.62-0.98)	0.035
	3	0.87 (0.82-0.92)	<0.001	0.92 (0.84-1.01)	0.063	0.85 (0.75-0.96)	0.007	0.84 (0.68-1.05)	0.127
	≥4	0.88 (0.84-0.92)	<0.001	0.97 (0.90-1.04)	0.325	0.84 (0.77-0.92)	<0.001	0.90 (0.77-1.06)	0.220
Coffee (cups/d)	Molel 1								
	0	1.00 (ref)		1.00 (ref)		1.00 (ref)		1.00 (ref)	
	1	0.90 (0.85-0.94)	<0.001	0.95 (0.88-1.02)	0.149	0.85 (0.76-0.94)	0.002	0.87 (0.73-1.05)	0.149
	2	0.96 (0.91-1.01)	0.091	0.97 (0.90-1.05)	0.441	0.99 (0.90-1.10)	0.895	0.85 (0.70-1.03)	0.087
	3	0.94 (0.89-1.00)	0.043	0.98 (0.90-1.07)	0.653	0.98 (0.87-1.10)	0.704	0.74 (0.59-0.94)	0.013
	≥4	1.12 (1.07-1.17)	<0.001	1.09 (1.02-1.17)	0.019	1.20 (1.09-1.31)	<0.001	1.12 (0.94-1.32)	0.207
	Molel 2								
	0	1.00 (ref)		1.00 (ref)		1.00 (ref)		1.00 (ref)	
	1	0.93 (0.89-0.98)	0.005	0.97 (0.90-1.05)	0.431	0.89 (0.80-0.99)	0.032	0.93 (0.77-1.11)	0.420
	2	0.98 (0.94-1.04)	0.535	0.98 (0.91-1.06)	0.623	1.04 (0.94-1.15)	0.506	0.87 (0.72-1.06)	0.168
	3	0.95 (0.90-1.01)	0.110	0.98 (0.90-1.07)	0.620	1.00 (0.89-1.13)	0.985	0.75 (0.59-0.95)	0.017
	≥4	1.05 (1.01-1.10)	0.031	1.02 (0.94-1.09)	0.679	1.13 (1.03-1.24)	0.012	0.99 (0.83-1.17)	0.871

Cox proportional hazards regression models adjusted for age, sex, ethnicity, education, and socioeconomic status in Model 1.

Model 2: adjusted for model one and smoking status, alcohol consumption frequency, physical activity frequency, BMI, and diet.

Table S4. HR (95% CI) of combined tea and coffee consumption with mortality

Tea	Coffee	All-cause mortality		Cancer mortality		CVD mortality		Respiratory mortality	
(c/d)	(c/d)	HR (95% CI)	*p*	HR (95% CI)	*p*	HR (95% CI)	*p*	HR (95% CI)	*p*
0	0	1.00 (ref)		1.00 (ref)		1.00 (ref)		1.00 (ref)	
	1	0.91 (0.76-1.08)	0.263	0.83 (0.63-1.11)	0.206	1.05 (0.74-1.5)	0.791	0.62 (0.31-1.24)	0.177
	2	0.92 (0.80-1.06)	0.238	0.90 (0.71-1.13)	0.348	1.10 (0.82-1.47)	0.534	0.75 (0.45-1.26)	0.278
	3	0.89 (0.78-1.03)	0.109	1.01 (0.82-1.26)	0.899	0.96 (0.72-1.29)	0.803	0.64 (0.38-1.07)	0.088
	≥ 4	0.92 (0.82-1.03)	0.135	0.99 (0.83-1.19)	0.922	1.06 (0.84-1.35)	0.627	0.76 (0.51-1.13)	0.170
1	0	0.93 (0.78-1.12)	0.443	0.90 (0.66-1.22)	0.494	1.26 (0.89-1.79)	0.196	0.79 (0.41-1.55)	0.497
	1	0.85 (0.71-1.02)	0.072	0.91 (0.68-1.20)	0.487	1.00 (0.70-1.44)	0.998	0.75 (0.39-1.43)	0.375
	2	0.93 (0.79-1.1)	0.423	1.18 (0.92-1.52)	0.182	0.96 (0.67-1.38)	0.840	0.68 (0.35-1.30)	0.240
	3	0.74 (0.62-0.89)	<0.001	0.80 (0.60-1.06)	0.123	1.01 (0.71-1.45)	0.943	0.54 (0.26-1.10)	0.090
	≥ 4	0.84 (0.73-0.98)	0.022	0.88 (0.70-1.11)	0.27	1.15 (0.86-1.54)	0.357	0.47 (0.26-0.86)	0.014
2	0	0.89 (0.77-1.02)	0.092	0.88 (0.70-1.11)	0.293	1.05 (0.79-1.4)	0.724	0.83 (0.50-1.36)	0.456
	1	0.80 (0.70-0.93)	0.003	0.96 (0.77-1.20)	0.715	0.79 (0.57-1.07)	0.130	0.64 (0.38-1.10)	0.103
	2	0.79 (0.69-0.91)	<0.001	0.79 (0.63-0.98)	0.034	1.04 (0.78-1.38)	0.790	0.65 (0.39-1.09)	0.104
	3	0.76 (0.65-0.9)	<0.001	0.78 (0.61-1.01)	0.059	0.92 (0.66-1.28)	0.611	0.34 (0.16-0.71)	0.004
	≥ 4	0.80 (0.70-0.93)	0.003	0.90 (0.72-1.13)	0.366	0.97 (0.72-1.3)	0.832	0.50 (0.28-0.86)	0.013
3	0	0.84 (0.73-0.96)	0.008	0.89 (0.72-1.1)	0.267	0.95 (0.73-1.25)	0.732	0.71 (0.44-1.13)	0.151
	1	0.81 (0.71-0.93)	0.003	0.94 (0.76-1.16)	0.562	0.89 (0.67-1.2)	0.449	0.72 (0.44-1.18)	0.193
	2	0.83 (0.72-0.95)	0.009	0.89 (0.72-1.12)	0.317	0.93 (0.69-1.25)	0.638	0.60 (0.35-1.03)	0.064
	3	0.76 (0.65-0.88)	<0.001	0.94 (0.75-1.19)	0.612	0.84 (0.61-1.16)	0.282	0.37 (0.19-0.72)	0.003
	≥ 4	0.80 (0.67-0.94)	0.008	0.76 (0.58-0.99)	0.047	0.82 (0.57-1.17)	0.262	0.85 (0.49-1.48)	0.556
≥ 4	0	0.83 (0.74-0.92)	<0.001	0.95 (0.80-1.13)	0.548	0.89 (0.70-1.12)	0.307	0.69 (0.47-1.01)	0.058
	1	0.76 (0.68-0.85)	<0.001	0.90 (0.75-1.08)	0.242	0.78 (0.61-0.99)	0.044	0.68 (0.45-1.02)	0.060
	2	0.82 (0.73-0.93)	<0.001	0.95 (0.79-1.14)	0.568	0.93 (0.72-1.20)	0.578	0.62 (0.40-0.95)	0.027
	3	0.80 (0.69-0.92)	0.002	0.93 (0.75-1.16)	0.518	0.91 (0.68-1.22)	0.516	0.70 (0.42-1.15)	0.156
	≥ 4	0.96 (0.85-1.08)	0.492	1.01 (0.83-1.23)	0.921	1.13 (0.87-1.46)	0.354	0.86 (0.56-1.32)	0.487

Cox proportional hazards regression models adjusted for age, sex, ethnicity, education, socioeconomic status, smoking status, alcohol consumption frequency, physical activity frequency, BMI, die, cholesterol-lowering medication, BP medication, insulin, depression, and tea or coffee intake in coffee or tea analysis. Abbreviations: c/d, cups/day; CVD,

cardiovascular diseases.

Table S5. HR (95% CI) of separate tea and coffee consumption with all-cause mortality by baseline characteristics

	Tea intake, cups/day					Coffee intake, cups/day				
Subgroup	0	1	2	3	≥4	0	1	2	3	≥4
Age										
< 60 years	1.00 (ref)	0.90 (0.78-1.02)	0.84 (0.74-0.94)	0.86 (0.77-0.97)	0.88 (0.80-0.96)	1.00 (ref)	0.94 (0.85-1.04)	1.01 (0.91-1.12)	0.95 (0.84-1.07)	1.00 (0.91-1.10)
≥60 years	1.00 (ref)	0.95 (0.87-1.03)	0.91 (0.85-0.98)	0.91 (0.85-0.98)	0.93 (0.88-0.99)	1.00 (ref)	0.93 (0.88-0.99)	0.98 (0.93-1.05)	0.95 (0.88-1.05)	1.08 (1.02-1.15)
Sex										
Female	1.00 (ref)	0.91 (0.81-1.02)	0.90 (0.82-0.99)	0.88 (0.80-0.97)	0.89 (0.82-0.96)	1.00 (ref)	0.92 (0.85-0.99)	1.01 (0.93-1.09)	0.94 (0.85-1.03)	1.07 (0.99-1.16)
Male	1.00 (ref)	0.96 (0.88-1.05)	0.89 (0.82-0.96)	0.91 (0.84-0.99)	0.93 (0.88-0.99)	1.00 (ref)	0.95 (0.89-1.01)	0.98 (0.92-1.05)	0.95 (0.88-1.03)	1.05 (0.99-1.12)
Ethnicity										
White	1.00 (ref)	0.93 (0.7-1.00)	0.90 (0.84-0.96)	0.90 (0.84-0.95)	0.91 (0.87-0.96)	1.00 (ref)	0.94 (0.89-0.99)	1.00 (0.95-1.05)	0.95 (0.90-1.02)	1.05 (1.00-1.11)
Others	1.00 (ref)	0.93 (0.69-1.25)	0.77 (0.59-1.01)	0.85 (0.65-1.13)	0.84 (0.65-1.08)	1.00 (ref)	0.83 (0.68-1.02)	0.95 (0.75-1.21)	0.77 (0.54-1.12)	1.23 (0.92-1.63)
Education										
Higher	1.00 (ref)	1.01 (0.87-1.16)	0.79 (0.69-0.91)	0.86 (0.75-0.98)	0.83 (0.74-0.93)	1.00 (ref)	1.06 (0.94-1.19)	1.01 (0.89-1.14)	0.99 (0.86-1.13)	1.02 (0.91-1.16)
Others	1.00 (ref)	0.91 (0.84-0.99)	0.92 (0.86-0.98)	0.91 (0.85-0.97)	0.93 (0.88-0.98)	1.00 (ref)	0.91 (0.86-0.96)	0.99 (0.94-1.05)	0.94 (0.88-1.00)	1.06 (1.01-1.12)
Socioeconomic status										
Higher	1.00 (ref)	0.94 (0.79-1.11)	0.95 (0.82-1.09)	0.97 (0.84-1.12)	0.90 (0.79-1.01)	1.00 (ref)	0.97 (0.86-1.10)	1.01 (0.89-1.15)	0.95 (0.83-1.10)	0.96 (0.85-1.10)
Intermediate	1.00 (ref)	0.98 (0.89-1.07)	0.91 (0.84-0.99)	0.89 (0.82-0.96)	0.93 (0.87-0.99)	1.00 (ref)	0.91 (0.85-0.97)	0.95 (0.88-1.01)	0.93 (0.86-1.01)	1.05 (0.98-1.12)
Lower	1.00 (ref)	0.85 (0.74-0.99)	0.82 (0.72-0.92)	0.87 (0.77-0.98)	0.88 (0.80-0.96)	1.00 (ref)	0.95 (0.86-1.05)	1.07 (0.97-1.19)	0.96 (0.85-1.09)	1.12 (1.02-1.23)
BMI										
<25 kg/m^2	1.00 (ref)	0.81 (0.59-1.11)	0.70 (0.53-0.92)	0.64 (0.48-0.85)	0.78 (0.63-0.96)	1.00 (ref)	0.79 (0.64-0.99)	0.99 (0.79-1.23)	0.68 (0.50-0.93)	1.18 (0.94-1.47)
25-29.9 kg/m^2	1.00 (ref)	1.02 (0.90-1.15)	0.95 (0.86-1.06)	0.94 (0.84-1.04)	0.95 (0.87-1.03)	1.00 (ref)	0.93 (0.85-1.02)	1.05 (0.96-1.15)	0.83 (0.89-1.10)	1.12 (1.03-1.23)
≥30 kg/m^2	1.00 (ref)	0.90 (0.83-0.99)	0.88 (0.81-0.95)	0.90 (0.83-0.97)	0.91 (0.85-0.96)	1.00 (ref)	0.95 (0.89-1.01)	0.96 (0.90-1.03)	0.94 (0.87-1.02)	1.03 (0.95-1.08)
Smoking status										
Never	1.00 (ref)	1.07 (0.96-1.21)	0.92 (0.83-1.02)	1.00 (0.91-1.11)	0.94 (0.87-1.02)	1.00 (ref)	0.94 (0.87-1.02)	1.08 (1.00-1.18)	0.97 (0.88-1.08)	1.10 (1.01-1.20)
Previous	1.00 (ref)	0.89 (0.80-0.98)	0.89 (0.81-0.97)	0.83 (0.76-0.91)	0.88 (0.82-0.95)	1.00 (ref)	0.92 (0.86-0.99)	0.97 (0.90-1.05)	0.94 (0.86-1.03)	0.99 (0.92-1.07)

Current	1.00 (ref)	0.82 (0.69-0.98)	0.84 (0.72-0.98)	0.82 (0.70-0.96)	0.91 (0.81-1.02)	1.00 (ref)	0.96 (0.84-1.10)	0.82 (0.71-0.94)	0.88 (0.76-1.02)	1.06 (0.95-1.18)
Alcohol consumption	1.00 (ref)					1.00 (ref)				
Low	1.00 (ref)	0.91 (0.80-1.05)	0.95 (0.85-1.06)	0.96 (0.86-1.07)	0.96 (0.88-1.04)	1.00 (ref)	0.97 (0.89-1.06)	0.98 (0.89-1.08)	0.97 (0.87-1.08)	1.08 (0.99-1.17)
Moderate	1.00 (ref)	0.96 (0.85-1.09)	0.89 (0.80-0.99)	0.90 (0.81-1.00)	0.93 (0.86-1.01)	1.00 (ref)	0.94 (0.87-1.03)	1.06 (0.97-1.16)	1.01 (0.91-1.11)	1.11 (1.02-1.20)
High	1.00 (ref)	0.93 (0.83-1.04)	0.85 (0.77-0.94)	0.84 (0.76-0.93)	0.86 (0.79-0.93)	1.00 (ref)	0.88 (0.80-0.96)	0.91 (0.83-0.99)	0.85 (0.77-0.94)	0.97 (0.88-1.06)
Physical activity										
Low	1.00 (ref)	0.92 (0.81-1.03)	0.84 (0.75-0.94)	0.81 (0.72-0.90)	0.85 (0.78-0.93)	1.00 (ref)	0.93 (0.85-1.02)	0.95 (0.86-1.04)	0.94 (0.85-1.05)	0.98 (0.89-1.07)
Moderate	1.00 (ref)	0.93 (0.85-1.03)	0.90 (0.83-0.97)	0.93 (0.86-1.01)	0.92 (0.86-0.98)	1.00 (ref)	0.95 (0.89-1.02)	1.01 (0.94-1.08)	0.93 (0.86-1.01)	1.09 (1.02-1.16)
High	1.00 (ref)	0.98 (0.81-1.20)	1.00 (0.85-1.19)	0.95 (0.80-1.12)	1.03 (0.90-1.18)	1.00 (ref)	0.87 (0.75-0.99)	1.02 (0.89-1.17)	1.04 (0.88-1.22)	1.11 (0.97-1.27)
Diet										
Unhealthy	1.00 (ref)	0.92 (0.84-1.01)	0.88 (0.82-0.96)	0.88 (0.81-0.95)	0.89 (0.83-0.95)	1.00 (ref)	0.89 (0.84-0.96)	0.98 (0.92-1.05)	0.93 (0.86-1.01)	1.02 (0.96-1.09)
Healthy	1.00 (ref)	0.96 (0.86-1.07)	0.90 (0.82-0.99)	0.92 (0.84-1.01)	0.95 (0.88-1.02)	1.00 (ref)	0.99 (0.92-1.07)	1.01 (0.93-1.09)	0.97 (0.88-1.06)	1.10 (1.02-1.19)
Cholesterol-lowering drug										
No	1.00 (ref)	0.93 (0.84-1.03)	0.88 (0.81-0.96)	0.89 (0.82-0.97)	0.89 (0.84-0.96)	1.00 (ref)	0.98 (0.91-1.05)	0.98 (0.91-1.05)	0.95 (0.88-1.04)	1.03 (0.97-1.11)
Yes	1.00 (ref)	0.94 (0.85-1.04)	0.90 (0.83-0.99)	0.90 (0.83-0.99)	0.93 (0.87-0.99)	1.00 (ref)	0.88 (0.82-0.95)	1.00 (0.93-1.08)	0.93 (0.85-1.02)	1.08 (1.00-1.16)
Antihypertensive drug										
No	1.00 (ref)	0.98 (0.88-1.08)	0.91 (0.83-0.99)	0.86 (0.78-0.94)	0.93 (0.86-0.99)	1.00 (ref)	0.95 (0.88-1.03)	0.97 (0.90-1.04)	0.92 (0.84-1.01)	1.08 (1.01-1.17)
Yes	1.00 (ref)	0.90 (0.82-0.99)	0.88 (0.81-0.95)	0.93 (0.85-1.00)	0.90 (0.84-0.96)	1.00 (ref)	0.92 (0.86-0.98)	1.01 (0.94-1.08)	0.97 (0.89-1.05)	1.03 (0.96-1.10)
Insulin										
No	1.00 (ref)	0.93 (0.86-0.99)	0.88 (0.82-0.93)	0.86 (0.81-0.92)	0.90 (0.85-0.94)	1.00 (ref)	0.92 (0.87-0.97)	0.98 (0.92-1.03)	0.95 (0.89-1.01)	1.07 (1.01-1.12)
Yes	1.00 (ref)	1.01 (0.78-1.31)	1.03 (0.83-1.28)	1.30 (1.06-1.59)	1.06 (0.89-1.25)	1.00 (ref)	1.10 (0.92-1.31)	1.16 (0.97-1.39)	0.92 (0.74-1.13)	0.94 (0.79-1.12)
Depression, n (%)										
No	1.00 (ref)	0.95 (0.88-1.02)	0.90 (0.84-0.96)	0.90 (0.85-0.96)	0.91 (0.87-0.96)	1.00 (ref)	0.94 (0.90-0.99)	1.00 (0.94-1.05)	0.98 (0.91-1.04)	1.07 (1.01-1.13)
Yes	1.00 (ref)	0.85 (0.68-1.06)	0.85 (0.70-1.02)	0.83 (0.69-0.99)	0.92 (0.80-1.05)	1.00 (ref)	0.84 (0.72-0.98)	0.98 (0.84-1.14)	0.73 (0.60-0.89)	0.97 (0.84-1.12)

Cox proportional hazards regression models adjusted for age, sex, ethnicity, education, socioeconomic status, smoking status, alcohol consumption frequency, physical activity frequency, BMI, die, cholesterol-lowering medication, BP medication, insulin, depression, and tea or coffee intake in coffee or tea analysis.

Table S6. HR (95% CI) of separate tea and coffee consumption with all-cause and cause-specific mortality when unadjusted for depression at recruitment

	All-cause mortality			Cancer mortality			CVD mortality			Respiratory mortality		
	Cases	HR (95% CI)	*p*	Cases	HR (95% CI)	*p*	Cases	HR (95% CI)	*p*	Cases	HR (95% CI)	*p*
Tea (cups/day)												
0	2875	1.00 (ref)		1184	1.00 (ref)		710	1.00 (ref)		212	1.00 (ref)	
1	1061	0.94 (0.87-1.00)	0.065	454	0.97 (0.87-1.08)	0.575	294	1.05 (0.92-1.21)	0.460	65	0.82 (0.62-1.08)	0.162
2	1779	0.89 (0.84-0.95)	<0.001	742	0.91 (0.83-0.99)	0.041	459	0.94 (0.83-1.06)	0.940	114	0.81 (0.64-1.02)	0.078
3	1867	0.90 (0.84-0.95)	<0.001	809	0.95 (0.86-1.04)	0.236	450	0.89 (0.79-1.00)	0.888	129	0.87 (0.70-1.10)	0.249
≥4	6084	0.91 (0.87-0.96)	<0.001	2724	1.00 (0.93-1.08)	0.949	1449	0.89 (0.81-0.98)	0.891	474	0.95 (0.79-1.14)	0.564
Coffee (cups/day)												
0	4148	1.00 (ref)		1738	1.00 (ref)		1014	1.00 (ref)		326	1.00 (ref)	
1	2431	0.93 (0.89-0.98)	0.006	1101	0.98 (0.90-1.05)	0.521	552	0.89 (0.80-0.99)	0.029	181	0.94 (0.78-1.13)	0.508
2	2396	0.99 (0.94-1.04)	0.715	1050	0.99 (0.92-1.07)	0.841	597	1.05 (0.94-1.16)	0.381	159	0.90 (0.74-1.10)	0.299
3	1534	0.95 (0.89-1.01)	0.072	694	0.99 (0.90-1.09)	0.833	382	0.99 (0.88-1.12)	0.854	90	0.77 (0.60-0.98)	0.031
≥4	3157	1.06 (1.00-1.11)	0.036	1330	1.04 (0.96-1.12)	0.381	817	1.13 (1.03-1.25)	0.014	238	1.03 (0.86-1.24)	0.716

Cox proportional hazards regression models adjusted for age, sex, ethnicity, education, socioeconomic status, smoking status, alcohol consumption frequency, physical activity frequency, BMI, die, cholesterol-lowering medication, BP medication, insulin, and tea or coffee intake in coffee or tea analysis. Abbreviations: c/d, cups/day; CVD, cardiovascular diseases. Abbreviations: CVD, cardiovascular diseases.

Table S7. HR (95% CI) of separate tea and coffee consumption with all-cause and cause-specific mortality after exclusion of individuals with CVD and cancer at recruitment (n = 98102)

	All-cause mortality			Cancer mortality			CVD mortality			Respiratory mortality		
	Cases	HR (95% CI)	*p*	Cases	HR (95% CI)	*p*	Cases	HR (95% CI)	*p*	Cases	HR (95% CI)	*p*
Tea (cups/day)												
0	1927	1.00 (ref)		814	1.00 (ref)		421	1.00 (ref)		148	1.00 (ref)	
1	737	0.94 (0.86-1.02)	0.139	328	0.99 (0.87-1.13)	0.930	182	1.07 (0.89-1.27)	0.483	43	0.73 (0.52-1.02)	0.068
2	1204	0.88 (0.82-0.95)	0.001	523	0.92 (0.82-1.03)	0.147	282	0.96 (0.82-1.12)	0.571	77	0.73 (0.55-0.97)	0.032
3	1255	0.89 (0.83-0.96)	0.002	558	0.95 (0.85-1.07)	0.400	288	0.96 (0.82-1.13)	0.616	80	0.73 (0.55-0.97)	0.031
≥4	3991	0.90 (0.85-0.96)	0.001	1809	0.99 (0.91-1.09)	0.866	850	0.92 (0.81-1.04)	0.173	311	0.85 (0.69-1.06)	0.147
Coffee (cups/day)												
0	2703	1.00 (ref)		1155	1.00 (ref)		586	1.00 (ref)		212	1.00 (ref)	
1	1638	0.93 (0.88-0.99)	0.031	746	0.97 (0.88-1.07)	0.491	332	0.90 (0.78-1.03)	0.118	130	0.99 (0.80-1.24)	0.940
2	1612	0.97 (0.91-1.03)	0.273	710	0.96 (0.87-1.05)	0.361	370	1.06 (0.93-1.21)	0.391	101	0.81 (0.64-1.04)	0.094
3	1038	0.93 (0.87-1.01)	0.066	477	0.97 (0.87-1.09)	0.635	242	1.03 (0.89-1.21)	0.672	58	0.70 (0.52-0.95)	0.020
≥4	2123	1.03 (0.97-1.10)	0.295	944	1.05 (0.96-1.15)	0.309	493	1.13 (1.00-1.29)	0.060	158	0.97 (0.78-1.22)	0.809

Cox proportional hazards regression models adjusted for age, sex, ethnicity, education, socioeconomic status, smoking status, alcohol consumption frequency, physical activity frequency, BMI, die, cholesterol-lowering medication, BP medication, insulin, depression, and tea or coffee intake in coffee or tea analysis. Abbreviations: c/d, cups/day; CVD, cardiovascular diseases. Abbreviations: CVD, cardiovascular diseases.

Supplementary Figure

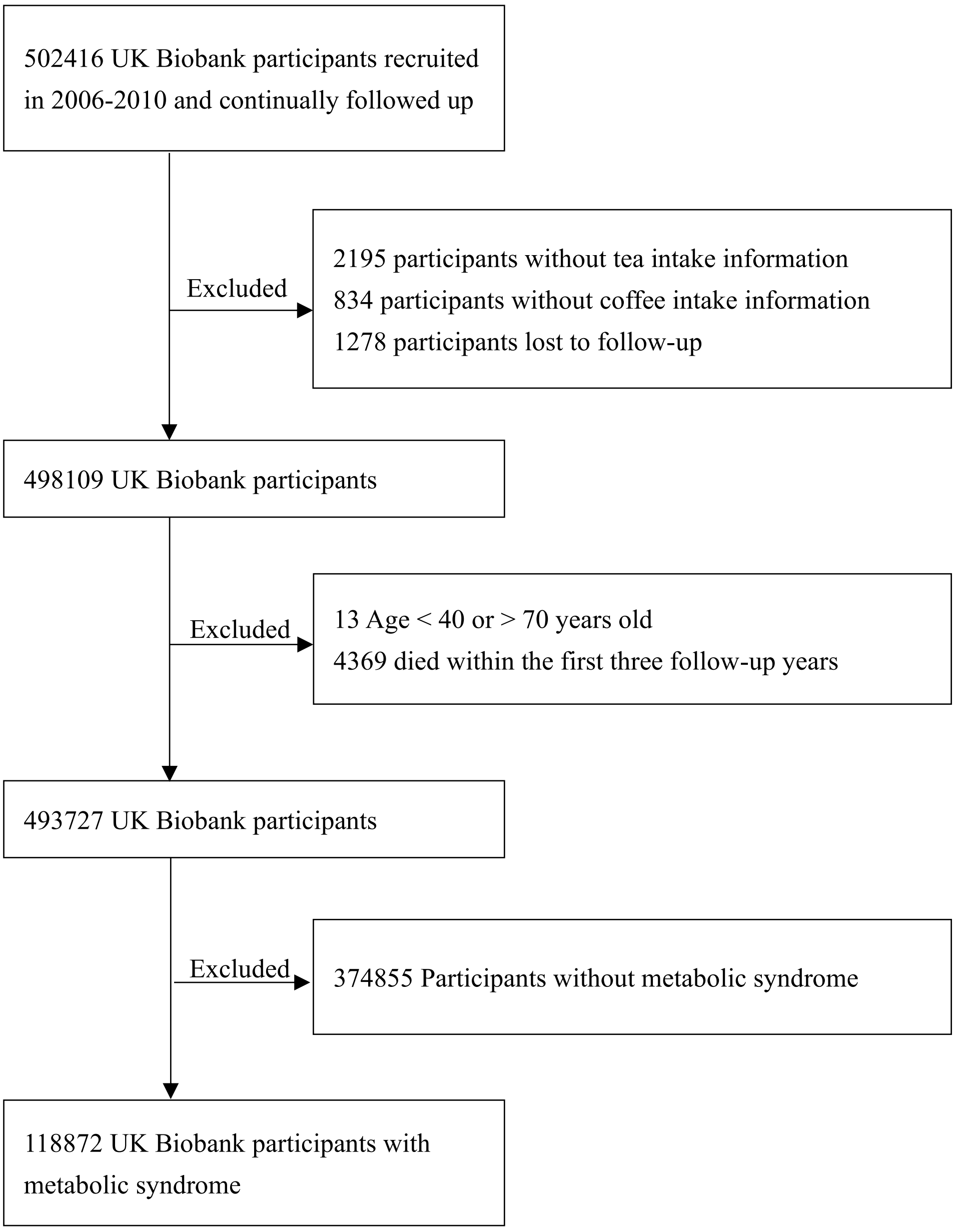

Fig. S1 Cohort exclusions of the study participants

Supplementary Methods

S1. Assessment of covariates

Age at recruitment: age when attended assessment center (years).

Sex: female, male.

Ethnicity: white ethnicity, other.

Education:

The original options were: College or University degree; A levels/AS levels or equivalent; O levels/GCSEs or equivalent; CSEs or equivalent; NVQ or HND or HNC or equivalent; Other professional qualifications eg, nursing, teaching; None of the above; Prefer not to answer.

In this study, we divided into five categories: (1) Higher: college or university; (2) Middle: A levels/AS levels or equivalent, O levels/GCSEs or equivalent; (3) Lower: CSEs or equivalent, NVQ, HND, HNC, or equivalent; (4) Vocational: professional qualifications, e.g., nursing, teaching; (5) Other.

Socioeconomic status: measured by Townsend deprivation index (TDI).

The TDI was calculated immediately prior to the participant joining UK Biobank. Based on the preceding national census output areas. Each participant is assigned a score corresponding to the output area in which their postcode is located. The TDI combined information on non-homeownership, household overcrowding, non-car ownership, and unemployment at recruitment, with a lower score indicating a higher socioeconomic status [1].

In the present study, it was divided into three groups: (1) Higher: lowest quintile; (2) Intermediate: quintiles 2-4; (3) Lower: highest quintile.

Body mass index (BMI):

BMI value is constructed from height and weight measured during the initial assessment centre visit (Kg/m^2).

Smoking status: never, previous, current smoking.

Alcohol intake frequency:

The touchscreen question “About how often do you drink alcohol?". The original options were: Daily or almost daily; Three or four times a week; Once or twice a week; One to three times a month; Special occasions only; Never; Prefer not to answer.

In the present study, it was divided into three groups: (1) Low: never, special occasions only; (2) Middle: one to three times a month; once or twice a week; (3) High: three or four times a week; daily or almost daily.

Physical activity frequency:

The touchscreen question “In a typical WEEK, on how many days did you do 10 minutes or more of moderate physical activities like carrying light loads, cycling at a normal pace? (Do not include walking)？ ” and “In a typical WEEK, how many days did you do 10 minutes or more of vigorous physical activity? (These are activities that make you sweat or breathe hard such as fast cycling, aerobics, heavy lifting)”. Units of measurement are days/week.

We add these two results and divide them by 2, and the final results are divided into three categories: (1) Low: ≤1; (2) Moderate: >1 and <=4 0.71; (3) High: >4 and < = 7.

Diet:

We defined the following 7 healthy eating behaviors: (1) Fruits ≥ 3 servings/day (Field ID: 1309, 1319); (2) Vegetables ≥ 3 servings/day (Field ID: 1289, 1299); (3) Fish ≥ twice a week (Field ID: 1329, 1339); (4) Whole grains ≥ 3 servings/day (Field ID: 1448,1438); (5) Processed meat ≤ once a week (Field ID: 1349); (6) Red meat ≤ twice a week (Field ID: 1369,1379,1389); (7) Refined grains ≤ 1 serving/day

(Field ID: 1458, 1468) [2].

Participants had at least the above four healthy eating habits, which are defined as a healthy diet, and others are defined as an unhealthy diet.

Medication history of anti-cholesterol drugs, antihypertensive drugs, insulin

The touchscreen question "Do you regularly take any of the following medications? (You can select more than one answer)". The original options were: Cholesterol lowering medication; Blood pressure medication; Insulin; Hormone replacement therapy; Oral contraceptive pill or minipill; None of the above; Do not know; Prefer not to answer.

Moreover, the touchscreen question "Do you regularly take any of the following medications? (you can select more than one answer)". The original options were: Cholesterol lowering medication; Blood pressure medication; Insulin; None of the above; Do not know; Prefer not to answer.

Medication history of anti-cholesterol drugs, anti-hypertensive drugs, or insulin selected for any of the options above was categorized as Cholesterol lowering medication (yes, no), Blood pressure medication (yes, no), and insulin (yes, no).

Depression

The depression was recorded using the ICD-10 code: F32. The code corresponds to a "depressive episode" [3]. We defined depressive episodes prior to recruitment, according to "Date F32 first reported"

S2. Assessment of missing values

The options for blank, "don't know" and "don't want to answer" were defined as missing values. For continuous variables, any missing values were replaced with the median value, such as body mass index and Townsend deprivation index; while for categorical variables, missing values were filled with the mode values.

	Missing value	
	Number	Proportion (%)
Education	1746	1.5
Ethnicity	231	0.2
Body mass index	402	0.3
Moderate activity frequency	8574	7.2
Vigorous activity frequency	8853	7.4
Physical activity frequency	4036	3.4
Smoking status	575	0.5
Alcohol consumption frequency	111	0.1
Townsend deprivation index	159	1.5

References

[1] Pei, Y.F.; Zhang, L. Is the Townsend Deprivation Index a Reliable Predictor of Psychiatric Disorders? *Biol. Psychiatry*. 2021, 89: 839–841.

[2] Wu E, Ni JT, Chen X, Zhu ZH, Xu HQ, Tao L, Xie T. Genetic risk, incident colorectal cancer, and the benefits of adhering to a healthy lifestyle: A prospective study using data from UK Biobank and FinnGen. *Front Oncol.* 2022, 12: 894086.

[3] Prigge R, Wild SH, Jackson CA. Depression, diabetes, comorbid depression and diabetes and risk of all-cause and cause-specific mortality: a prospective cohort study. *Diabetologia*. 2022, 65(9): 1450-1460.
